# Model for Coordination of Microtubule and Actin Dynamics in Growth Cone Turning

**DOI:** 10.3389/fncel.2018.00394

**Published:** 2018-10-31

**Authors:** Erin M. Craig

**Affiliations:** Department of Physics, Central Washington University, Ellensburg, WA, United States

**Keywords:** cytoskeleton organization, computational modeling, neuronal cytoskeleton, microtubule dynamics, growth cone guidance

## Abstract

In the developing nervous system, axons are guided to their synaptic targets by motile structures at the axon tip called growth cones, which reorganize their cytoskeleton in order to steer in response to chemotactic cues. Growth cone motility is mediated by an actin-adhesion “clutch” mechanism, in which mechanical attachment to a substrate, coupled with polarized actin growth, produces leading-edge protrusion. Several studies suggest that dynamic microtubules (MTs) in the growth cone periphery play an essential role in growth cone steering. It is not yet well-understood how the MT cytoskeleton and the dynamic actin-adhesion clutch system are coordinated to promote growth cone navigation. I introduce an experimentally motivated stochastic model of the dynamic reorganization of the growth cone cytoskeleton in response to external guidance cues. According to this model, asymmetric decoupling of MTs from actin retrograde flow leads to a local influx of MTs to the growth cone leading edge, and the leading-edge MT accumulation is amplified by positive feedback between MTs and the actin-adhesion clutch system. Local accumulation of MTs at the leading edge is hypothesized to increase actin adhesion to the substrate, which attenuates actin retrograde flow and promotes leading-edge protrusion. Growth cone alignment with the chemotactic gradient is predicted to be most effective for intermediate levels of sensitivity of the adhesion strength to the presence of leading-edge MTs. Quantitative predictions of the MT distribution and the local rate of retrograde actin flow will allow the hypothetical positive feedback mechanism to be experimentally tested.

## Introduction

**Figure 1:**
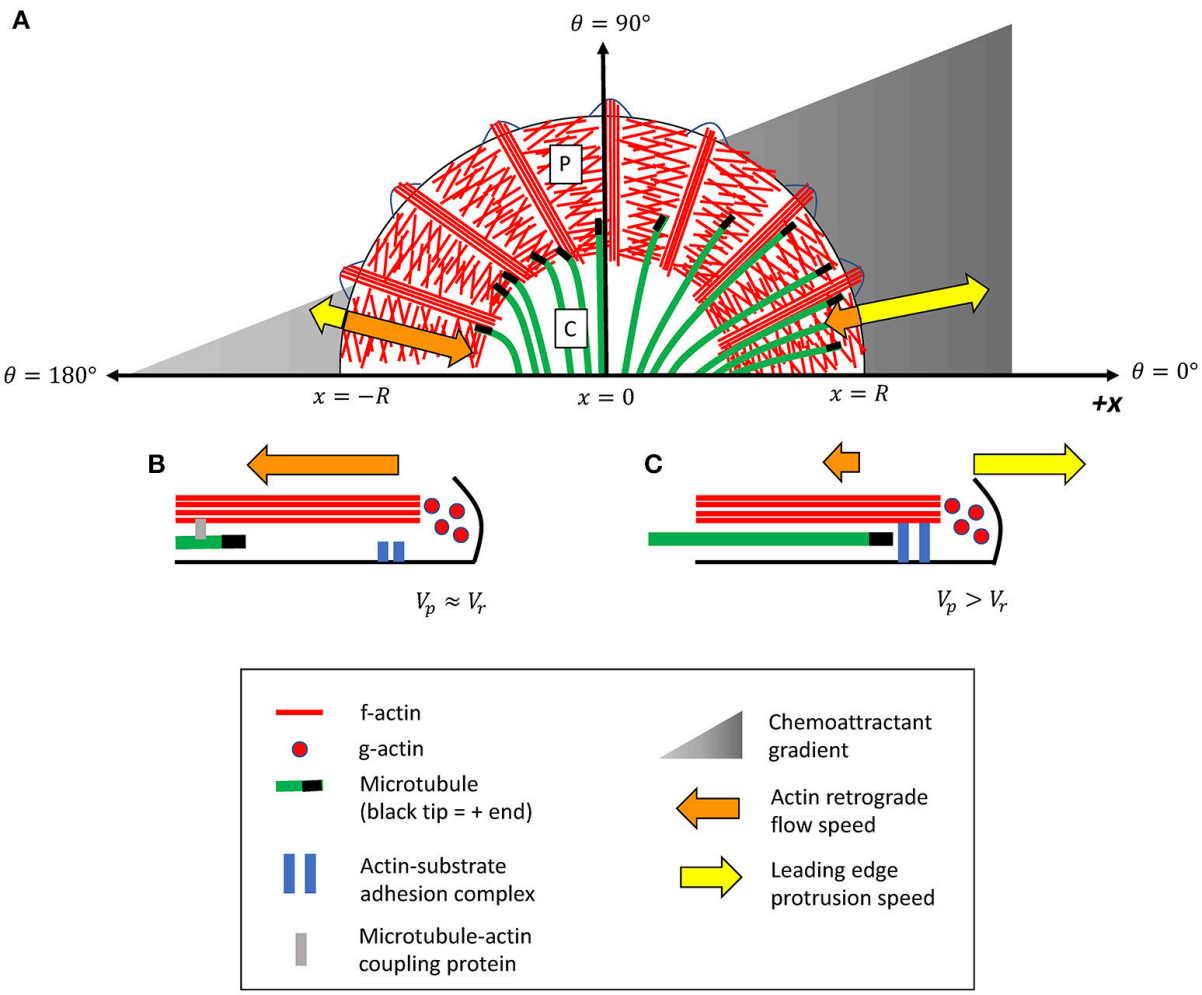
Illustrative schematic of feedback model for MT-actin-adhesion coordination in growth cone turning. **(A)** The peripheral (P) domain contains a flat branched network of lamellipodial actin, interspersed by parallel actin bundles called filopodia. Dynamic microtubules (MTs) originating in the central (C) domain occasionally extend into the peripheral (P) domain. A chemoattractant gradient induces asymmetric decoupling of MTs from actin retrograde flow (Equation 5), causing MTs to accumulate in the P domain on one side of the growth cone more than the other. Presence of leading-edge MTs promotes local “clutch engagement” (Equation 3), producing attenuated retrograde flow and increased leading edge protrusion on this side of the growth cone. **(B)** Side-view schematic illustrating MT-actin-adhesion interactions on the side of the growth cone exposed to a lower concentration of the attractive external signal. MTs on this side of the growth cone have a high probability of coupling to actin retrograde flow and being translocated away from the leading edge. The f-actin network is in a “treadmilling” state in which most leading-edge actin polymerization is canceled by actin retrograde flow. **(C)** Schematic of MT-actin-adhesion interactions on the side of the growth cone exposed to a higher concentration of external signal. MTs on this side of the growth cone are more likely to decouple from actin and extend into the P domain. Leading-edge MTs promote actin adhesion to the substrate, slowing the rate of actin retrograde flow and promoting local cell protrusion.

Specialized sensory-motile structures called growth cones at the tips of growing axons lead neuronal pathfinding during nervous system development. Growth cones rely on spatiotemporal coordination of the leading-edge cytoskeleton to translate external chemical cues into a mechanical response (Dent and Gertler, 2003; Bard et al., 2008; Coles and Bradke, 2015). Growth cones are composed of a central region (C domain) filled with organelles and microtubules, and a peripheral region (P domain) composed of a flat dense network of actin filaments (Figure 1) (Lowery and Van Vactor, 2009). When actin adhesion to the substrate is weak, the f-actin network undergoes retrograde flow away from the leading edge, driven by a combination of leading edge membrane tension and myosin contractile forces in the transitional zone (Lin et al., 1996; van Goor et al., 2012). When the retrograde flow speed matches the rate of leading edge polymerization, a condition known as “treadmilling,” the continual turnover of actin in the absence of leading-edge protrusion can be likened to a vehicle with an unengaged clutch, expending energy without driving growth cone motility (Mitchison and Kirschner, 1988; Lin and Forscher, 1995; Jay, 2000; Bard et al., 2008). When adhesion to the substrate increases, thus “engaging the clutch,” retrograde flow of actin is slowed, allowing leading-edge polymerization to drive cell protrusion.

While many of the mechanical components of the actin “clutch” mechanism for leading-edge protrusion have been identified and characterized (Lowery and Van Vactor, 2009; Craig et al., 2012; Kerstein et al., 2015), an ongoing challenge is to elucidate the mechanism for growth cone steering in response to external signals. Several lines of evidence suggest that growth cone turning relies on coordination between f-actin and dynamic microtubules (MTs) from the C domain that explore the growth cone periphery (Geraldo and Gordon-Weeks, 2009). In *Aplysia* bag growth cones, mechanical coupling between MTs and actin serves as a barrier to MT entry into the P domain by causing MTs to undergo retrograde translocation at the same rate as actin (Forscher and Smith, 1988; Schaefer et al., 2002, 2008; Burnette et al., 2007). A number of putative MT-actin linkers have been identified (Rodriguez et al., 2003; Cammarata et al., 2016), but the precise nature of MT-actin coupling in growth cones remains under investigation. During adhesion-mediated growth cone guidance, microtubules asymmetrically invade the peripheral domain of the growth cone in the direction of the turn (Tanaka and Kirschner, 1995; Aih and Suter, 2008). Fluorescent speckle microscopy (FSM) imaging of MTs suggests that this reorganization takes place primarily through uncoupling from actin retrograde flow rather than from changes in polymerization rates (Aih and Suter, 2008). When microtubules are prevented from interacting with f-actin in the P domain, either through global stabilization or depolymerization of MTs, growth cones lose their ability to turn in response to guidance cues (Williamson et al., 1996; Challacombe et al., 1997; Buck and Zheng, 2002).

Several lines of experimental investigation over the last several decades have demonstrated that axons are sensitive to external gradients of molecular guidance cues (Rosoff et al., 2004; Vitriol and Zheng, 2012; Goodhill, 2016). *In-vitro* assays have characterized the alignment of axonal outgrowth along gradients of guidance cue molecules (Bicknell et al., 2018), and axons *in vitro* can respond to even shallow gradients of guidance cues such as nerve growth factor (Bicknell et al., 2018). Patterns of molecular guidance cue gradients are present in the developing nervous system (Kennedy et al., 2006; Sloan et al., 2015), and genetic manipulation of guidance cue gradients *in vivo* is associated with axonal miswiring (Chédotal and Richards, 2010; Kang et al., 2018). An important unresolved challenge is to quantitatively characterize the mechanisms of cytoskeletal reorganization downstream of external guidance cues that promote leading edge growth cone protrusion in a direction aligned with external signaling gradients.

Theoretical models have provided insight into several aspects of axon guidance and growth cone steering. Agent-based biased turning models simulate axon guidance in the presence of external guidance cues, using Bayesian statistics to predict neuronal response to external gradients in order to elucidate quantitative limitations of gradient sensing mechanisms (Mortimer et al., 2009; Catig et al., 2015). Other studies have investigated how the Rac1-stathmin-MT signaling pathway regulates MT polarization and leading-edge protrusion in growth cones (Mahajan and Athale, 2012; Zeitz and Kierfeld, 2014; Xu and Bressloff, 2015). In (Craig et al., 2012), we used a mathematical continuum model to characterize the forces involved in the f-actin treadmill in the growth cone P domain in order to elucidate the relative contributions of myosin contractile stress and membrane tension force in driving actin retrograde flow. In contrast to these models, the present study will investigate how interactions between MTs and f-actin in the P domain impact the balance of forces that govern leading edge protrusion driven by actin polymerization. A goal of this study is to test hypothetical mechanisms for how guidance cue gradients govern the dynamic mechanical feedback between MTs and f-actin in the P domain in order to promote asymmetric leading-edge membrane protrusion.

Here, I introduce a minimal model for coordination between the growth cone's actin-based “engine and clutch” and microtubule-based “steering” systems in response to a gradient of attractive guidance cues. I use agent-based simulations to predict the direction of the initial leading edge actin-based protrusion, which is the first characteristic stage of growth cone re-organization preceding axon outgrowth (Lowery and Van Vactor, 2009). I hypothesize that growth cone steering is coordinated through a positive feedback mechanism in which an attractive guidance cue decreases the coupling of MTs to actin retrograde flow, allowing MTs to invade the periphery of the growth cone. Increased presence of MTs near the leading edge, in turn, promotes actin adhesion to the substrate, thereby “engaging the clutch” to enable local cell protrusion. Attenuated actin retrograde flow has the additional effect of allowing even more MTs to invade the leading edge, thus amplifying the growth cone's response to an external signal. I develop a mathematical description of this hypothetical feedback mechanism, and demonstrate that dynamic coordination between MTs, actin, and adhesions promotes growth cone alignment along guidance cue gradients.

## Model

The mathematical model described here is predicated on several key assumptions:

1. **MTs transiently couple to actin retrograde flow and are translocated away from the leading edge**. This assumption is based on experimental observations that approximately 65% of MTs in the growth cone periphery move retrogradely at speeds matching actin retrograde flow (Schaefer et al., 2002, 2008). Although the mechanism for MT-actin coupling has not been fully established, a number of putative MT-actin linkers have been identified (Rodriguez et al., 2003; Cammarata et al., 2016).
2. **The likelihood of MT-actin mechanical coupling decreases in the presence of an attractive chemical cue**. This assumption is supported by experimental observations that guidance cues can differentially regulate MT-actin coupling in different regions of a growth cone (Aih and Suter, 2008). I assume that the attractive guidance cue acts primarily at the growth cone leading edge, but that downstream signals could travel in the retrograde direction with f-actin flow, thus also affecting MT-actin coupling away from the leading edge.
3. **MTs in the growth cone periphery promote actin adhesion to the substrate, allowing actin polymerization to drive local cell protrusion**. This assumption is based on observations that microtubule accumulation at an adhesion site, and attenuation of actin retrograde flow, both accompany adhesion-mediated growth cone guidance (Aih and Suter, 2008). A plausible explanation is that MTs act as a track for the delivery of cell adhesion molecules to the leading edge of the growth cone, thus promoting asymmetric adhesion along the axis of migration.

I use a modified version of the reaction-drift equations introduced by Dogterom and Leibler (1993) to describe the density of microtubule tips along a one-dimensional slice of a growth cone in the radial direction (Figure 1):

(1) $$\begin{array}{rcl} \partial_{t}p_{+} & = & -f_{\pm }p_{+}+f_{\mp }p_{-}-\left(v_{+}-p_{r}v_{r}\right)\partial_{r}p_{+} \end{array}$$

(2) $$\begin{array}{rcl} \partial_{t}p_{-} & = & f_{\pm }p_{+}-f_{\mp }p_{-}+\left(v_{-}+p_{r}v_{r}\right)\partial_{r}p_{-} \end{array}$$

Here, *p*_+_ and *p*_−_ describe the density of growing and shrinking MTs, respectively. MTs polymerize at a speed of *v*_+_ and depolymerize at a speed of *v*_−_, switching between states of growth and shrinking at rates of *f*_±_ and *f*_∓_, respectively. I assume that MTs have a probability, *p*_*r*_, to mechanically link to the f-actin network and be pulled away from the leading edge at the speed of actin retrograde flow, *v*_*r*_. Based on a combination of polymerization and translocation, the tip of a growing MT moves toward the leading edge at an average speed of *v*_+_−*p*_*r*_*v*_*r*_, whereas the tip of a shrinking MT moves away from the leading edge at an average speed of *v*_−_+*p*_*r*_*v*_*r*_.

The retrograde flow speed, *v*_*r*_, is a dynamic variable determined by the balance of forces acting on the f-actin network in the P domain: *F* = (ξ_0_+ξ_*M*_*p*)*v*_*r*_. Here, *F* represents the sum of forces driving retrograde flow, ξ_0_ is the viscous resistance to actin flow in the absence of leading edge MTs, *p* is the fraction of MTs that extend past the 75% line of the growth cone (*r*>0.75*R*), and ξ_*M*_ characterizes the additional viscous resistance promoted by leading edge MTs. This force-balance relationship can be expressed as:

(3) $$\begin{array}{r} \frac{v_{r}}{v_{0}}=\frac{1}{1+\beta p} \end{array}$$

where we define $v_{0}=\frac{F}{\xi_{0}}$ as the maximum retrograde flow speed, and $\beta =\frac{\xi_{M}}{\xi_{0}}$ as a measure of the sensitivity of the substrate adhesion to leading-edge MTs. According to the actin-adhesion “clutch” mechanism, leading edge cell protrusion is determined by the difference between leading edge actin polymerization speed, *v*_*p*_, and retrograde flow of actin at speed *v*_*r*_:

(4) $$\begin{array}{r} v_{cell}=v_{p}-v_{r} \end{array}$$

Note that if the actin polymerization speed matches the maximum actin retrograde flow speed (*v*_*p*_ = *v*_0_), such that the system is in a “treadmilling” steady-state in the absence of leading-edge MTs, then combining Equations (3, 4) yields: $\frac{v_{cell}}{v_{0}}=1-\frac{v_{r}}{v_{0}}=\frac{\beta p}{1+\beta p}$, indicating a saturating relationship between leading edge protrusion speed and the density of MTs at the leading edge.

The growth cone is described with a simplified two-dimensional geometry of a semi-circle, with angular coordinates shown in Figure 1. The intensity of the chemoattractant signal increases linearly in the x-direction indicated in Figure 1 at a rate of α, such that the likelihood of MTs coupling to actin decreases as a function of the x-position. I assume that the MT-actin coupling probability in the P domain is a function of the external signal strength at the leading edge, such that a MT entering the P domain from the C domain has a coupling probability that depends only on θ and not on the radial coordinate of the MT tip. The MT-actin coupling probability at the growth cone leading edge can be expressed in terms of angular coordinates within the growth cone as *p*_*r*_ = *p*_*r*0_−∝*R*cosθ, where and *R* is the growth cone radius and *p*_*r*0_ is the coupling probability at θ = 90. Defining the dimensionless parameter $A=\frac{\alpha R}{p_{r0}}$, the MT-actin coupling probability can be written as:

(5) $$\begin{array}{r} p_{r}=p_{r0}\left(1-A\cos\theta \right) \end{array}$$

Taken together, Equations (1–5) describe a mechanism in which leading edge protrusion depends sensitively on the probability, *p*_*r*_, for MTs to mechanically couple to actin retrograde flow. When *p*_*r*_ is low, MTs invade the leading edge and promote substrate adhesion, which in turn allows more MTs to accumulate at the leading edge, amplifying the growth cone's response to spatial variation in the external signal.

The model parameters characterizing microtubule and actin dynamics can be estimated based on experimental measurements (Table 1). I set *p*_*r*0_ = 0.5 as a reference value for this study, consistent with the order of magnitude of typical estimated frequencies of MT coupling to retrograde flow in growth cones (Schaefer et al., 2002, 2008). The two remaining unconstrained parameters *A* and β encapsulate key assumptions of the model: *A* characterizes the steepness of the external guidance cue gradient, which determines the degree to which MT-actin coupling varies across the growth cone. When *A* = 0, MT-actin coupling probability is uniform across the growth cone, while values of *A* approaching 1 correspond to maximum asymmetry in MT-actin coupling. For example, for *A* = 0.9, 5% of MTs couple to actin in the direction of the turn (θ = 0), in contrast to 95% of MTs coupled to actin flow on the opposite side (θ = 180) (Equation 5). The tunable parameter β characterizes the degree to which MTs at the leading edge promote actin adhesion to the substrate. To determine how these parameters impact the ability of a growth cone to turn in response to an external guidance signal, agent-based simulations based on Equations (1–5) are performed for discrete angular coordinates from θ = 0 to θ = 180, yielding predictions of the steady-state values of *p*, *v*_*r*_, and *v*_*cell*_ at each angle. A net protrusion vector can be calculated based on a vector sum of local protrusion vectors, in order to determine the net direction of growth cone protrusion, θ_*p*_, for a given combination of *A* and β. Simulation methods are described in more detail in the Supplemental Information.

**Table 1:** Estimates of dynamic parameters for MTs and actin in growth cones based on fluorescent imaging of microtubules and f-actin in the growth cones of *Aplysia* bag cell neurons.

**Parameter**	**Value**	**Source**
Catastrophe frequency, *f*_±_	$0.61\;\overset{-1}{\min}$	Burnette et al., 2007
Rescue frequency, *f*_∓_	$1.79\;\overset{-1}{\min}$	Burnette et al., 2007
MT polymerization speed, *v*_+_	6.0 μ*m*/min	Burnette et al., 2007
MT depolymerization speed, *v*_−_	9.6 μ*m*/min	Burnette et al., 2007
Maximum actin retrograde flow speed, *v*_0_	5 μ*m*/min	Burnette et al., 2007; Aih and Suter, 2008
Actin polymerization speed, *v*_*p*_	5 μ*m*/min	Mitchison and Kirschner, 1988

*Unless otherwise specified, the values listed here are used in all simulations*.

## Results

**Figure 2:**
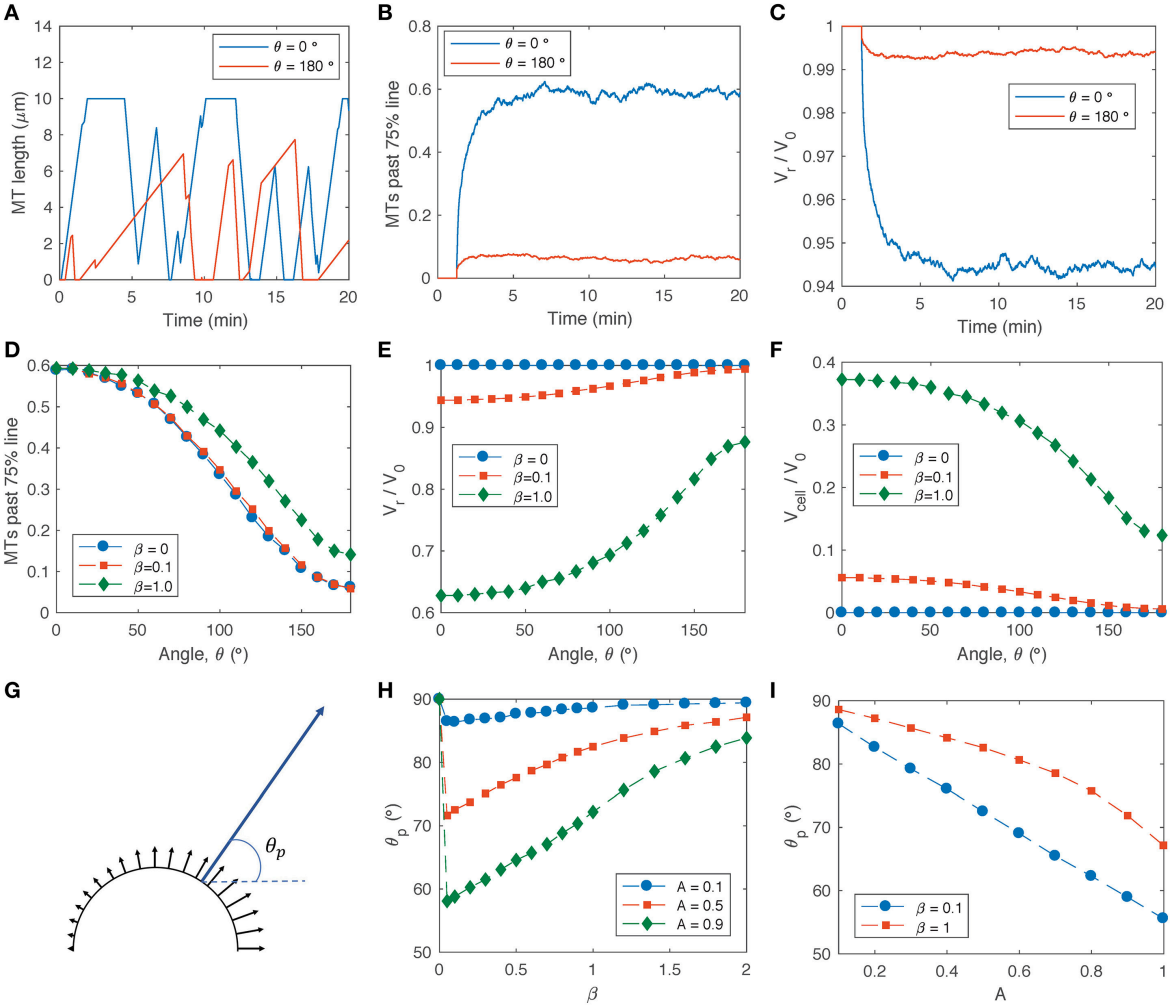
MT dynamics and actin retrograde flow in the growth cone P domain based on the MT-actin-adhesion feedback model (Equations 1–5). Parameters listed in Table 1 are used unless otherwise noted. **(A)** Sample simulations of MT length vs. time for two individual MTs in different regions of a growth cone in the presence of an attractive guidance cue that increases in the x-direction in Figure 1 (such that MT-actin coupling probability is lowest for θ = 0 and highest for θ = 180, according to Equation 5). Parameters: *A* = 0.9, β = 0.1. **(B)** Fraction of MTs that extend past the 75% line (*r* = 0.75*R*) as a function of time, based on a simulation of 1000 MTs for each angular coordinate. **(C)** Actin retrograde flow speed as a function of time, for the same simulations as in **(B)**. **(D)** Average steady-state fraction of MTs past 75% line as a function of angle within the growth cone, for signal gradient parameter *A* = 0.9 and several values of the adhesion sensitivity parameter β. **(E)** Average steady-state actin retrograde flow speed as a function of angle within the growth cone, for *A* = 0.9 and several values of β. **(F)** Average steady-state local cell protrusion speed as a function of angle within the growth cone, for *A* = 0.9 and several values of β. **(G)** Schematic of local cell protrusion vectors based on the relative magnitudes and directions from the simulations in **(F)** (black arrows), and the net protrusion vector calculated as a vector sum of each of the local protrusion vectors (blue arrow). The net protrusion angle, θ_*p*_, is labeled. **(H)** Net protrusion angle, θ_*p*_, as a function of β, for several values of *A*. **(I)** Net protrusion angle, θ_*p*_, as a function of *A* for several values of β.

Simulations based on Equations (1–5) demonstrate that asymmetric decoupling of MTs from actin, followed by promotion of actin adhesion at the site of MT invasion, allows a growth cone to re-orient its direction of leading-edge protrusion in response to an external guidance cue (Figure 2). On the side of the growth cone exposed to a stronger signal (θ = 0), MTs are in a state of unbounded growth and frequently reach the growth cone leading edge (Figure 2A, blue line). On the opposite side of the growth cone (θ = 180), MTs are more frequently coupled to actin retrograde flow, such that the growth and shrinking speeds of the MT tips are augmented by retrograde translocation and the MTs are in a state of bounded growth (Figure 2A, red line). Consequently, a larger number of MTs accumulate on the side of the growth cone with the stronger signal (Figure 2B). Actin retrograde flow is attenuated in the regions of the growth cone with higher MT accumulation (Figure 2C, blue line), thus “engaging the clutch” for local cell protrusion, while actin continues to “treadmill”—turning over unproductively—on the opposite side of the growth cone (Figure 2C, red line).

To investigate the reorganization of the P domain cytoskeleton in the presence of a steep signal gradient (*A* = 0.9), I obtained steady-state averages of the fraction of MTs past the 75% line (Figure 2D), actin retrograde flow speed (Figure 2E) and local cell protrusion speed (Figure 2F) for a range of angular coordinates across the growth cone. For the limiting case in which actin adhesion to the substrate is not sensitive to the presence of leading-edge MTs (β = 0), decoupling of MTs from actin retrograde flow produces an asymmetric MT distribution across the growth cone (Figure 2D, blue circles), although it does not produce asymmetric actin retrograde flow (Figure 2E, blue circles). For non-zero values of the adhesion sensitivity parameter, β, MT excursion into the P domain is amplified by a positive feedback loop in which leading-edge MTs promote adhesion activation, and the resulting attenuation in retrograde flow allows more MTs to accumulate (Figures 2D,E, green diamonds). While higher values of β produce faster overall rates of local cell protrusion (Figure 2F, green diamonds), lower β values produce slow protrusion on one side of the growth cone and actin treadmilling on the other side (Figure 2F, red squares).

The direction, θ_*p*_, of net growth cone protrusion can be determined based on a sum of local protrusion vectors (Figure 2G), where θ_*p*_ = 0 corresponds to protrusion aligned with the external signal gradient and θ_*p*_ = 90 corresponds to cell protrusion orthogonal to the signal gradient and aligned with the original axis of the growth cone. The strongest turning response (smallest θ_*p*_) occurs for very small non-zero values of β (Figure 2H), and θ_*p*_ approaches 90 with increasing β, because higher adhesion sensitivity promotes clutch engagement on both sides of the growth cone. For a given value of β, the net protrusion angle decreases with increasing signal gradient, *A* (Figure 2I).

## Concluding remarks

The model presented here demonstrates a hypothetical mechanism for growth cone steering in which asymmetric MT invasion of the P domain promotes adhesion-mediated growth cone turning. Simulations of this model recapitulate experimentally observed features of cytoskeletal re-organization in response to guidance cues, including MT invasion of the growth cone periphery in the direction of the turn, and attenuation of retrograde flow accompanied by additional MT accumulation at sites of increased substrate adhesion. The simulated degree of turning is directly correlated to the steepness of an external signal gradient, in agreement with *in-vitro* observations of axonal alignment with chemotactic gradients (Bicknell et al., 2018). A prediction of this model is that growth cone turning is most effective for an intermediate level of sensitivity of the actin-adhesion “clutch” to the presence of MTs at the leading edge (characterized by our model parameter β): If adhesion increases in response to MTs, and attenuation of retrograde flow further promotes the local accumulation of MTs, this allows the growth cone to amplify its mechanical response to an external gradient. However, if the adhesion activation mechanism is overly sensitive to MTs, causing the “clutch” to engage too easily, actin retrograde flow will be slowed on both sides of the growth cone, reducing the effectiveness of growth cone pathfinding. An ongoing challenge will be to identify possible molecular or signaling mechanisms that could account for the hypothetical relationship between exploratory MTs and substrate adhesion.

The deliberately simple framework of this model is intended to synthesize experimentally motivated hypotheses about MT and f-actin coordination in growth cones, in order to provide a cohesive description of MT-actin feedback in growth cone steering supported by numerical simulations. A central assumption of the model is that the probability of mechanical coupling between MTs and f-actin in the P domain is directly proportional to the density of attractive guidance cues at the growth cone leading edge. Validation and refinement of this assumption may emerge from ongoing experimental investigation of the linking proteins involved in MT coupling to actin retrograde flow and the biochemical signaling pathways involved in regulating these interactions. This model framework could be expanded to investigate the role of molecular motors in microtubule redistribution during growth cone turning (Turney and Bridgman, 2005; Grabham et al., 2007; Nadar et al., 2012; Kahn and Baas, 2016), or to investigate mechanochemical regulation of adhesion and force production in growth cone motility (Craig et al., 2015; Kerstein et al., 2015).

## Author contributions

The author confirms being the sole contributor of this work and has approved it for publication.

### Conflict of interest statement

The author declares that the research was conducted in the absence of any commercial or financial relationships that could be construed as a potential conflict of interest.
