# Supplementary material for: Model for Coordination of Microtubule and Actin Dynamics in Growth Cone Turning
Source: Front Cell Neurosci. 2018 Oct 31;12:394. doi: 10.3389/fncel.2018.00394 (PMC6225807; doi:10.3389/fncel.2018.00394)
Supplement: Supplementary file 1 [file Data_Sheet_1.PDF]

**Supplemental materials:**

**Computational simulation methods**

The following protocol was used to simulate MT distributions and corresponding actin retrograde flow speeds and leading-edge protrusion speeds based on the model described by Equations (1)-(5):

i. For a given angular coordinate, the simulation is initiated with *N* microtubules of length $L_i = 0$, and each MT is randomly assigned one of four possible states:
**State 1:** A growing MT that is not mechanically coupled to actin
**State 2:** A shrinking MTs not mechanically coupled to actin
**State 3:** A growing MT that is mechanically coupled to actin
**State 4:** A shrinking MT that is mechanically coupled to actin

ii. In each time step of the simulation, the length of each MT is updated according to:

$$L_i = L_i + \tilde{v}\Delta t \qquad \text{(S1)}$$

where the time step duration, $\Delta t$, is set small enough that the typical length excursion during a single time step is much smaller than the growth cone radius ($\tilde{v}\Delta t \ll R$). The rate of change in the length, $\tilde{v}$, is defined for States 1-4 in the following way:

$$\tilde{v} = \begin{pmatrix} v_+ \\ -v_- \\ v_+ - v_r \\ -(v_- + v_r) \end{pmatrix} \qquad \text{(S2)}$$

Here, $v_+$ and $v_-$ are the average speed of MT growth and shrinking respectively. The speed of actin retrograde flow, $v_r$, is calculated according Equation (3) in the main text. If the length of a MT exceeds the growth cone radius, $R$, it is set to $L_i = R$. If the length of a MT drops below zero in a time-step calculation, the length is set to $L_i = 0$.

iii. In each time step, every MT can transition between states according to the following transition probability matrix:

$$\Pi = \begin{pmatrix} \Pi_{11} & \Pi_{12} & \Pi_{13} & \Pi_{14} \\ \Pi_{21} & \Pi_{22} & \Pi_{23} & \Pi_{24} \\ \Pi_{31} & \Pi_{32} & \Pi_{33} & \Pi_{34} \\ \Pi_{41} & \Pi_{42} & \Pi_{43} & \Pi_{44} \end{pmatrix}$$

$$= \begin{pmatrix} 1 - f_\pm\Delta t - f_{da}\Delta t & f_\pm\Delta t & f_{da}\Delta t & 0 \\ f_\mp\Delta t & 1 - f_\mp\Delta t - f_{da}\Delta t & 0 & f_{da}\Delta t \\ f_{ad}\Delta t & 0 & 1 - f_\pm\Delta t - f_{ad}\Delta t & f_\pm\Delta t \\ 0 & f_{ad}\Delta t & f_\mp\Delta t & 1 - f_\mp\Delta t - f_{ad}\Delta t \end{pmatrix} \qquad \text{(S3)}$$

Here, $\Pi_{ij}$ represents the probability for a MT to transition from State *i* to State *j* during a single time step. The parameters $f_\pm$ and $f_\mp$ represent rates of catastrophe and rescue, respectively. The rate at which a MT attaches to actin retrograde flow is $f_{da}$, and the rate at which a MT that is attached to actin detaches is $f_{ad}$. The attachment and detachment rates are related as: $f_{ad} = \left(\frac{1-p_r}{p_r}\right) f_{da}$, where $p_r$ is the probability for a MT to be mechanically attached to actin. The attachment probability is calculated in terms of the angular coordinate of the MT, according to Equation (5) in the main text.
